# Non-Expanding Plaster

**Published:** 1904-08

**Authors:** P. B. M’Cullough

**Affiliations:** Philadelphia


### NON-EXPANDING PLASTER.

BY P. B. M'CULLOUGH, D.D.S., PHILADELPHIA.

WHAT degree of credit for priority attaches to the following original experiments with plaster the writer is unable to state. It would seem that credit for fixing definitely the treatment of the water might be assumed. The writer first saw in a pamphlet advertising Dodel elevators, entitled "Pointers for Dentists," the statement that plaster mixed with lime-water would not shrink.

Following this the writer, as a test to prevent "expansion," used lime-water that had been prepared for domestic use. This water had been filtered, then boiled, and when cold lime had been added to saturation.

A mix of plaster was made with this water and poured in glass tubes; that the tubes did not crack after several months was evidence that the plaster had not expanded. As the stock of lime-water was reduced by its use in the laboratory, water as drawn from the supply pipes was added as required. Later a second test was made, and all the tubes cracked within twelve minutes. That the first tests were made with boiled water and the second with water without boiling made it clear that the water must be boiled to prevent expansion; otherwise the addition of lime makes no perceptible difference. In order to establish definitely wherein the secret lay, the following tests were made. A quantity of water was filtered, then boiled for fifteen minutes, when cold unslaked lime was added in excess of saturation. When the solution had become clear, it was decanted into a smaller bottle, that the sediment might not be stirred up in handling. With this water a mix of plaster was made of the ordinary consistency and placed in a glass tube, tapping the tube on the bench to insure a solid filling. After several hours it was soaked in water as drawn from the spigot, with no change; it was then placed in a vulcanizer resting on a flask above the water and subject to 320° F. for an hour, this test showed the tube without crack.

The second test consisted in adding a little salt to the lime-water. In the third test a little water from the supply pipes was added to the lime-water. For the fourth test plain boiled water, cold, was used. In each case the tubes cracked within twelve minutes.

A solution of gum-arabic, made with plain water, was used, and the test showed the tube without crack. This last experiment was not further tested, because more plaster could be mixed with the lime-water than with the gum-arabic water, hence with the latter the plaster was less dense. It was further thought that the free solubility of the gum-arabic in water might cause the plaster to soften in vulcanizing. The use of lime-water in all of my plaster work, particularly for vulcanite dentures, has been attended with such uniformly gratifying results that I would consider a return to the former practice a retrograde change. For impressions I use "impression plaster." For the model and for flasking I use the "slow-setting." This latter has the advantage of becoming harder than the "medium," these being the three grades of dental plaster with which the writer is familiar. That the reader may have all the information in my possession bearing upon this valuable discovery the following data is submitted:

Doctor X. Dodel,
San Francisco, Cal.:

My dear Doctor,—I see in your "Pointers for Dentists," No. 3, 1903, on page 4, "To prevent shrinkage in plaster, mix with lime-water." I will very much appreciate your stating whether there is any special way for preparing the lime-water.

Yours very truly,
P. B. McCullough.

February 19, 1904.

DR. P. B. McCULLOUGH,
Philadelphia, Pa.:

DEAR DOCTOR,—Lime-water is a saturated solution of calcium hydrate, and you will find its preparation in the American Pharmacopœia. Use it *cold* instead of common water. It is inexpensive and you can get it at any drug store.

Yours very truly,
(Signed) X. DODEL.

February 24, 1904.

It will be seen that in the "Pointer" referred to the word "shrinkage" is used, and likewise in my letter. This is unsatisfactory, as the problem with the dentist has always been to prevent expansion.

It must be observed, however, that lime-water bought at a drug-store is presupposedly made with distilled water, and when so prepared it would serve the purpose, but as there can be no certainty that bought lime-water is always so prepared, no dentist using it can be said to do so intelligently without the above information, particularly as the writer is informed by a druggist that not one per cent. of the lime-water sold is made with boiled water, and that at most only filtered water is used.

